# Functional analysis of isoprenoid precursors biosynthesis by quantitative metabolomics and isotopologue profiling

**DOI:** 10.1007/s11306-019-1580-8

**Published:** 2019-08-21

**Authors:** Sara Castaño-Cerezo, Hanna Kulyk-Barbier, Pierre Millard, Jean-Charles Portais, Stéphanie Heux, Gilles Truan, Floriant Bellvert

**Affiliations:** 1 LISBP, Université de Toulouse, CNRS, INRA, INSA, Toulouse, France; 2 MetaToul-MetaboHUB, National Infrastructure of Metabolomics and Fluxomics, Toulouse, France

**Keywords:** Systems biology, Mass spectrometry, Isoprenoids, Isotope labeling experiments

## Abstract

**Introduction:**

Isoprenoids are amongst the most abundant and diverse biological molecules and are involved in a broad range of biological functions. Functional understanding of their biosynthesis is thus key in many fundamental and applicative fields, including systems biology, medicine and biotechnology. However, available methods do not yet allow accurate quantification and tracing of stable isotopes incorporation for all the isoprenoids precursors.

**Objectives:**

We developed and validated a complete methodology for quantitative metabolomics and isotopologue profiling of isoprenoid precursors in the yeast *Saccharomyces cerevisiae*.

**Methods:**

This workflow covers all the experimental and computational steps from sample collection and preparation to data acquisition and processing. It also includes a novel quantification method based on liquid chromatography coupled to high-resolution mass spectrometry. Method validation followed the Metabolomics Standards Initiative guidelines.

**Results:**

This workflow ensures accurate absolute quantification (RSD < 20%) of all mevalonate and prenyl pyrophosphates intermediates with a high sensitivity over a large linear range (from 0.1 to 50 pmol). In addition, we demonstrate that this workflow brings crucial information to design more efficient phytoene producers. Results indicate stable turnover rates of prenyl pyrophosphate intermediates in the constructed strains and provide quantitative information on the change of the biosynthetic flux of phytoene precursors.

**Conclusion:**

This methodology fills one of the last technical gaps for functional studies of isoprenoids biosynthesis and should be applicable to other eukaryotic and prokaryotic (micro)organisms after adaptation of some organism-dependent steps. This methodology also opens the way to ^13^C-metabolic flux analysis of isoprenoid biosynthesis.

**Electronic supplementary material:**

The online version of this article (10.1007/s11306-019-1580-8) contains supplementary material, which is available to authorized users.

## Introduction

Isoprenoids form one of the most abundant and diverse family of biological molecules on Earth (Buckingham 1993). They are produced by all organisms, from prokaryotes to eukaryotes, and are involved in a wide range of biological activities, including maintenance of membrane fluidity, defense against fungi and other pathogens, signaling, growth regulators, attractants for pollinators, or precursors for the synthesis of hormones, bile acids and sterols (Goldstein and Brown 1990; Liao et al. 2016). Moreover, isoprenoids possess strong biological activities against human diseases and are now used as anti-cancers, anti-inflammatories, antioxidants, antibacterial and health supplements (Barbieri et al. 2017; Cho et al. 2017; Gill et al. 2016; Rodriguez-Concepcion et al. 2018; Wang et al. 2005). Furthermore, an alteration of the isoprenoid levels in human cells has been related to several human diseases (Mullen et al. 2016; Pelleieux et al. 2018). The broad range of commercial applications of isoprenoids in agriculture, cosmetics and health has driven intense efforts to develop cost-effective and sustainable manufacturing processes in biotechnology (Ko et al. 2018; Lauersen 2018; Withers and Keasling 2007; Zhang et al. 2017). Comprehensive understanding of the operation and regulation of isoprenoids biosynthesis is thus key in a broad range of fundamental and applicative fields, including systems biology, medicine and biotechnology.

In *Saccharomyces cerevisiae*, as in many other eukaryotes and prokaryotes, isoprenoids biosynthesis starts with the condensation of two molecules of acetyl-CoA via the mevalonate pathway, which comprises six enzymatic steps to produce isopentenyl diphosphate (IPP), the common precursor of all isoprenoids (Katsuki and Bloch 1967; Lange et al. 2000). IPP is isomerized into dimethylallyl diphosphate (DMAPP), which is then condensed with IPP to generate geranyl pyrophosphate (GPP). Longer prenyl pyrophosphates are built from the condensation of IPP onto each intermediate, giving farnesyl pyrophosphate (FPP) from GPP and geranylgeranyl pyrophosphate (GGPP) from FPP. Evidently, accurate quantification of the mevalonate and prenyl pyrophosphates intermediates in biological samples is necessary to characterize the operation of isoprenoids biosynthesis, and ultimately understand its control and regulation. Only a few methods have been reported for the analysis of isoprenoids precursors (Supporting Table S-1). Virtually all available methods are limited to the quantification of only a few specific intermediates, and none of these studies aimed at measuring their labeling patterns. This would open the way towards isotope labeling experiments (ILEs) for metabolic flux analyses of isoprenoids biosynthesis. The most advanced method developed by Henneman et al. allows the quantification of all the intermediates in a single run by liquid chromatography coupled to mass spectrometry (LC–MS) (Henneman et al. 2008). Analysis of isoprenoids precursors could be improved further by reducing time and cost constrictions (e.g. caused by the preparation of internal standards by enzymatic or chemical synthesis for absolute quantification), as well as reducing the risk of instrument damage due to trimethylamine (e.g. it makes virtually impossible future analyses in positive mode without very extensive cleaning) (Rütters et al. 2000). Finally, sampling and sample preparation procedures have never been investigated nor optimized in detail despite being of utmost importance to ensure accurate measurement of metabolite concentrations and isotopic tracer incorporation (Bolten et al. 2007; Millard et al. 2014).

In this work, we present a complete framework for functional analysis of isoprenoids biosynthesis in yeast. This workflow covers all the experimental and computational steps, with in-depth evaluation of sampling, sample preparation, data acquisition and data processing procedures. We demonstrate that our workflow ensures accurate absolute quantification of all the intermediates in a single analysis and can also measure their incorporation of tracer—i.e. isotopologue distributions—in isotopic labeling experiments.

## Experimental section

### Strains and cultivations

All strains used in this study were derived from *Saccharomyces cerevisiae* CEN-PK2.1C (www.uni-frankfurt.de/fb15/mikro/euroscarf). To test the performance of the analytical method, we constructed two strains (S023 and S037) with higher levels of isoprenoid precursors than the wild-type strain. Both had integrated one extra copy of HMG1t and ERG20 under TDH3 and PGK promoters respectively. Furthermore, in S023 and S037 the GGPP synthase from *Xanthophyllomyces dendrorhous* (CrtE) was expressed constitutively (TEF1p) in a centromeric plasmid. Additionally, the strain S023 also bears one integrated copy of the phytoene synthase (converts GGPP to phytoene) from *Pantoea ananatis* (CrtB) under the transcriptional control of the PDC1p. This strain was used to test if the GGPP pool was consumed. The genotypes of the different strains are detailed in Supporting Table S-2.

All strains were grown in a modified Verduyn synthetic complete media containing glucose (111 mM), NH_4_Cl (75 mM), KH_2_PO_4_ (22 mM), MgSO_4_ (0.4 mM) and CSM Leu^−^ (ForMedium LTD, Hunstaton, England) at pH 5.0 (Verduyn et al. 1992).

For metabolomics experiments, an initial OD_600nm_ of 0.007 was used to inoculate 50 mL of Verduyn medium Leu^−^ and cells were grown at 28 °C with an agitation of 220 rpm (MaxQ, ThermoFisher) until harvest at mid-exponential phase. To prepare the ^13^C internal standard used throughout this study, strain S037 was pre-cultured in 20 mL of Verduyn medium without any amino acids and 55 mM U-^13^C-glucose. This preculture was used to inoculate 600 mL of the same medium at an initial OD of 0.05. Cells were harvested at mid-exponential phase.

For isotope labeling experiments (ILEs), 200 mL of the same medium was inoculated at an initial OD of 0.007. When the OD was around 3, 10 mL of culture were harvested (time zero) and centrifuged (3000×*g*, 28 °C, 5 min), and cells were resuspended in the same medium with 55 mM of U-^13^C-glucose (99% ^13^C-purity, Eurisotop, Saint-Aubin, France) instead of unlabeled glucose. Samples were harvested at times 0, 1, 2, 5, 10, 15, 30, 45, 60, 90 and 120 min after the switch of label input.

### Sampling and sample preparation

Intracellular metabolites were sampled by fast filtration (Kiefer et al. 2007; Millard et al. 2014). Briefly, 10 mL of broth were filtered through 0.45 μm Sartolon polyamide (Sartorius, Goettingen, Germany) and washed with 5 mL of fresh culture medium (without glucose). The filters were rapidly plunged into liquid nitrogen and then stored at −80 °C until extraction. Intracellular metabolites were extracted by incubating filters in closed glass tubes containing 5 mL of an isopropanol/H_2_0 NH_4_HCO_3_ 100 mM (50/50) mixture at 70 °C for 10 min. For absolute metabolite quantification 50 µL of ^13^C internal standard were added to each extract. Cellular extracts were cooled on ice and sonicated during 1 min. Cell debris was removed by centrifugation (5000×*g*, 4 °C, 5 min). Supernatants were evaporated overnight (SC110A SpeedVac Plus, ThermoFisher, Waltham, MA, USA), resuspended in 200 μL of methanol: NH_4_OH 10 mM (7:3) at pH 9.5 and stored at − 80 °C until analysis.

### LC–MS analyses of metabolic intermediates

Analyses were carried out on a LC–MS platform composed of a Thermo Scientific™ Vanquish™ Focused UHPLC Plus system with DAD, coupled to a Thermo Scientific™ Q Exactive™ Plus hybrid quadrupole-Orbitrap™ mass spectrometer (ThermoFisher).

Analysis of isoprenoids precursors was performed on a Thermo Scientific™ Hypersil C18 GOLD™, 3 µm, and 2.1 × 100 mm column. The column was kept at 25 °C and the flow rate was set to 0.3 mL/min during first 2 min and 0.4 mL/min for the rest chromatographic run. The solvent system consisted of (A) 20 mM NH_4_FA, pH 9.5, in water and (B) 20 mM NH_4_FA, pH 9.5, in 9:1 (v/v) acetonitrile–water, with the following gradient: 0–12 min from 100% A to 100% B, 5.5 min kept with 100% B and within 0.5 min the return to the initial condition and 4 min equilibration of the column. The injection volume was 10 µL.

Mass detection was carried out in a negative electrospray ionization (ESI) mode. The settings of the mass spectrometer were as follows: spray voltage 3.2 kV, capillary and desolvation temperature were 350 and 400 °C respectively, maximum injection time 200 ms. Nitrogen was used as sheath gas (50 a.u.) and auxiliary gas (15 a.u.). The automatic gain control (AGC) was set at 106 and resolution at 70,000 from m/z 100 to 700. MS analyses were performed by targeted selected ion monitoring (tSIM) mode with 0.5 m/z isolation window for SIM and ± 10 ppm for inclusion tolerances. tSIM MS scans the targeted masses in different time segments selected based on the retention times of the analytes. Data acquisition was performed using Thermo Scientific Xcalibur software.

Internal standards obtained from uniformly ^13^C-labeled extracts from yeast were used to enable isotope-dilution mass spectrometry (IDMS) (Wu et al. 2005) for the identification and quantification of following compounds: MEV (CAS number 150-97-0), M5P (CAS number 1189-94-2), M5PP (CAS number 4872-34-8), IPP (CAS number 358-71-4) and DMAPP (CAS number 358-72-5) (isobaric compound not separated by the chromatographic method), GPP (CAS number 763-10-0), FPP (CAS number 13058-04-3), and GGPP (CAS number 6699-20-3). All metabolites were identified following minimum standards for level 1 identification (Sumner et al. 2007). Calibration mixtures (prepared at concentrations from 0.08 nM to 10 µM of each isoprenoid precursor) were used to construct calibration curves enabling to determine the concentration of each compound in the samples to be measured.

### Quantification of phytoene

10 mL of yeast culture were harvested, centrifuged, and washed with 1 mL of MilliQ water. Cell pellets were freeze-dried and stored at − 80 °C until extracted. 0.11 nmol/mg DCW of β-apocarotenal was added to the dried cells, and phytoene was extracted with glass beads and 500 µL of acetone by three rounds of agitation of 20 s at 0.05 m/s with FastPrep FP120 (ThermoFisher). The acetone phase was transferred to a new tube and the extraction was repeated twice. Acetone extracts were pooled, centrifuged, dried under nitrogen flux and resuspended in 100 μL of acetone for HPLC analysis.

Analyses were carried out on a Thermo Scientific™ Vanquish™ Focused UHPLC Plus system with DAD. 5 µL of the extraction was injected in a column YMC carotenoid (100 × 2.0 mm and 3 µm particle size) equipped with a precolumn (100 × 2.0 mm and 3 µm particle size). The mobile phases used to separate and quantify phytoene and β-apocarotenal from the ergosterol and derivatives consisted in a mixture of A methanol/water (95:5) and B dichloromethane. The flow was 0.25 mL/min with the following gradient: 0–0.1 min 5% B, 0.1–0.5 min 20% B, 0.5–2 min 60% B, 2–5 min 80% B, 5–8 min 80% B and 8–11 min 5% B. Absorbance from 210 to 600 nm was followed during all the run with a data collection rate of 2 Hz and response time of 2 s. Phytoene was quantified by its absorbance at 286 nm and β-apocarotenal at 478 nm. Reference wavelength (600 nm) was subtracted for each of the wavelengths used for metabolite quantification.

### Data processing and statistical analysis

MS data were processed using TraceFinder v3.2 (ThermoFisher), with a tolerance of 5 ppm to extract exact masses. In ^13^C-ILEs, isotopologue distributions were quantified from mass fractions after correction for the presence of all naturally occurring isotopes and isotopic purity of the tracer (99%) using IsoCor v2.0.4 (Millard et al. 2019), which ensures accurate correction of high-resolution isotopic data. IsoCor also calculates the mean ^13^C-enrichment (*E*) from the isotopologue distributions using the following formula:

1 $$E = \frac{{\mathop \sum \nolimits_{i = 1}^{n} i \cdot M_{i} }}{n}$$

where *M*_*i*_ is the proportion of the isotopologue with *i* ^13^C atoms for a metabolite containing *n* carbon atoms. IsoCor is freely available at https://github.com/MetaSys-LISBP/IsoCor and its documentation at https://isocor.readthedocs.io.

Labeling dynamics between metabolites and strains were compared as detailed in (Kiefer et al. 2015). Briefly, time-course ^13^C-enrichments were first fitted using the following logistic model:

2 $$E\left( t \right) = \frac{{k \cdot y_{0} \cdot e^{t \cdot T} }}{{k + y_{0} \cdot e^{t \cdot T} - y_{0} }}$$

Half time *T*_*50*_, which corresponding to the time needed to exchange half of ^12^C atoms of a metabolite pool by ^13^C atoms, were then calculated using the following equation:

3 $$T_{50} = \frac{1}{T} \cdot \ln \frac{{k - y_{0} }}{{y_{0} }}$$

For metabolomics experiments, standard deviations were calculated from three independent biological replicates, each with two technical replicates. For isotopic profiling, standard deviations were calculated from two independent biological experiments, with one technical replicate. For each intermediate, a two-sided Welch’s *t* test was used to assess the significance of differences of concentrations and labeling dynamics observed between the strains.

## Results and discussion

### LC–HRMS analysis of isoprenoids precursors

Metabolic intermediates of the isoprenoid biosynthetic pathway (MEV, M5P, M5PP, IPP, DMAPP, GPP, FPP and GGPP) were analyzed by reverse phase chromatography and detected by high-resolution mass spectrometry (HRMS) operating in negative mode. The MS acquisition method was optimized to enhance sensitivity and selectivity by using the tSIM mode. Selectivity was enhanced further by setting the resolution of the Orbitrap cell to 70,000, which was sufficient to avoid mass interference with the analytes of interest, even in complex cellular extracts. Finally, the mass scan range was limited to 600 m/z (from 100 to 700 m/z) to avoid systematic under representation of heavier isotopologues (Su et al. 2017) and ensure accurate metabolomics and isotopic results. Chromatographic separation of phosphorylated intermediates was improved by replacing metal tubing of the LC system by more inert peek tubing (Tuytten et al. 2006). Peaks tailing was reduced further by increasing the pH of the eluent and of the resuspension buffer to 9.5 to reduce adsorption on the stainless steel injector (Tuytten et al. 2006).

**Fig. 1:**
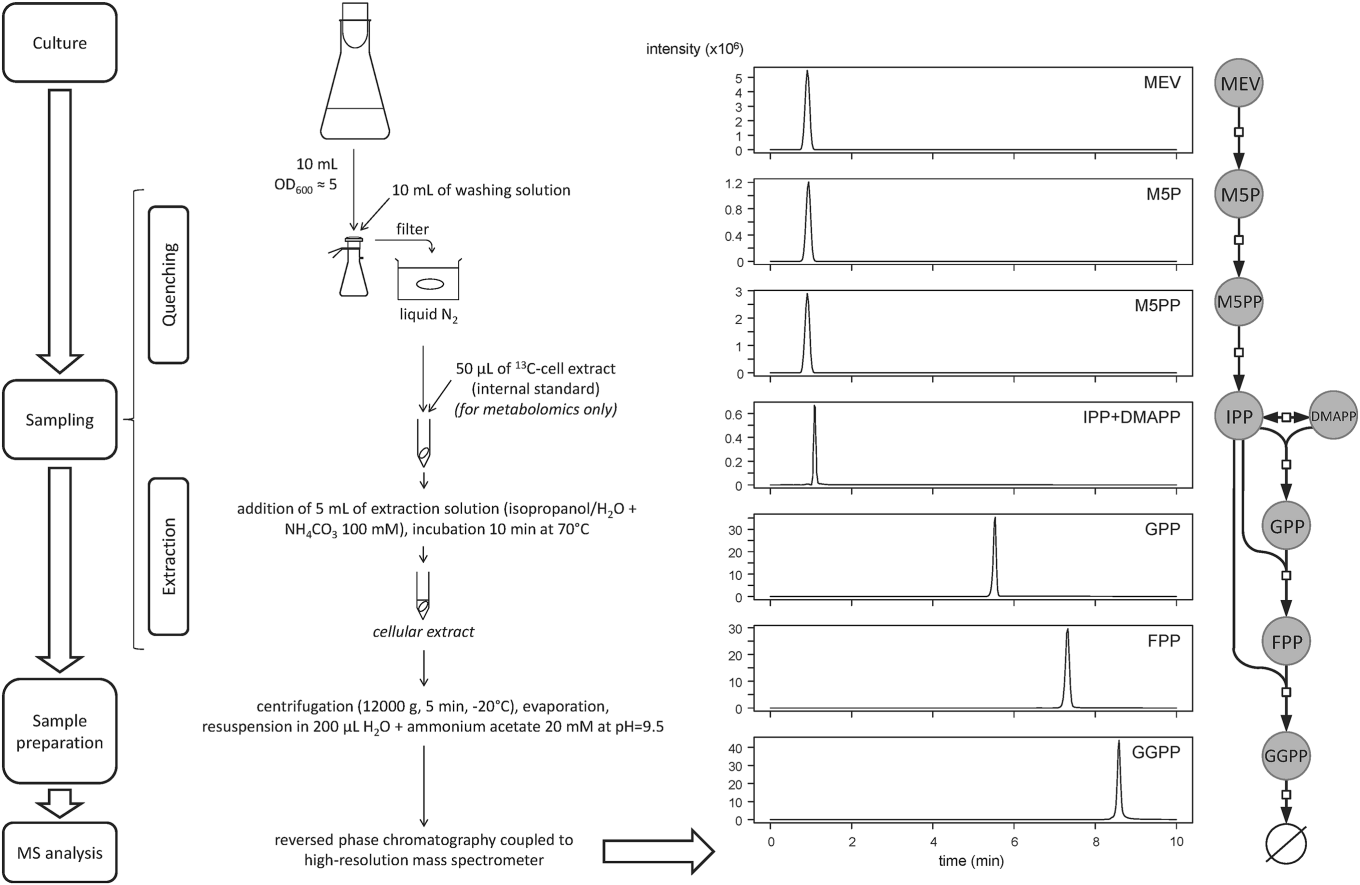
Experimental workflow for quantification and isotopologue profiling of isoprenoids precursors in yeast. Cells are quenched in liquid nitrogen after fast filtration, and intracellular metabolites are extracted by incubating the filters during 10 min in 5 mL of isopropanol/H_2_O 100 mM NH_4_HCO_3_ (1:1) at 70 °C. The extract is centrifuged, dried, resuspended in 200 µL of methanol/NH_4_OH 10 mM (7:3) at pH 9.5, and analyzed by LC–HRMS. Chromatograms of commercial standards (at a concentration of 5 µM) prepared in a biological matrix are shown on the right

Solutions of commercial standards at 5 µM were used to determine the retention time of each compound (Table 1). A chromatogram demonstrating the separation of the isoprenoids precursors in a biological matrix is shown in Fig. 1.

**Table 1:** Method validation for quantitative metabolomics of isoprenoids precursors

Metabolite	Retention time (min)	Exact mass (m/z)	LOD (pmol)	Linear range (pmol)
MEV	1.2	147.06628	0.1	1.5–50
M5P	1.2	227.03261	0.02	0.1–50
M5PP	0.9	306.99894	0.02	0.1–50
IPP	1.2	244.99855	0.01	0.2–50
DMAPP	1.2	244.99855	0.05	0.1–50
GPP	5.7	313.06115	0.01	0.5–50
FPP	7.4	381.12375	0.01	0.2–50
GGPP	8.7	449.18635	0.1	0.8–50

*LOD* limit of detection, expressed in pmol of compound injected on the column

Except IPP and DMAPP which are isobaric compounds and could not be separated by the chromatographic method, all compounds could be detected with a high selectivity. We verified that M5PP does not contribute to the signal of M5P (e.g. due to in-source fragmentation and loss of a phosphate group) and observed a low interference (< 10%). Similarly, M5P does not interfere with measurement of MEV (< 10%).

### Workflow for functional analysis of isoprenoids biosynthesis

With this LC–MS method in hand, we then focused on optimizing the sampling procedure, which is of utmost importance to obtain a reliable picture of the metabolome (Bolten et al. 2007; Millard et al. 2014). Intracellular metabolites of *S. cerevisiae* were collected by fast filtration to ensure rapid quenching of metabolism while reducing the concentrations of glucose, amino acids and salts that would impair both chromatographic separation and MS ionization. The extraction procedure was optimized to maximize metabolite recovery (amount recovered and reproducibility). Four different extraction procedures were tested. The first one was a cold extraction, acetonitrile/methanol/water (4:4:2) at − 20 °C during 1 h. This method has been described to be very efficient and specially for triphosphate compounds (Rabinowitz and Kimball 2007). Secondly, we tested three different protocols already used for isoprenoid extractions in different organisms. All of them use extraction solutions with basic pH and the extractions are performed at high temperature. The first one has already been used for the fission yeast *Schizosaccharomyces pombe.* This protocol consists in an extraction with isopropanol/H_2_O + 100 mM NH_4_HCO_3_ (1:1) at 70 °C during 15 min (Takami et al. 2012). We also tested if the extraction could be enhanced for *S. cererevisiae* using other organic solvents: ethanol/H_2_O + 100 mM NH_4_HCO_3_ (3:1) at 70 °C during 15 min (Tong et al. 2008), or butanol/ethanol/H_2_O + 100 mM NH_4_HCO_3_ (4:5:11) extraction at 70 °C during 15 min (Tong et al. 2005). The most efficient extraction solutions were isopropanol/H_2_O + 100 mM NH_4_HCO_3_ (1:1) and butanol/ethanol/H_2_O + 100 mM NH_4_HCO_3_ (4:5:11), but the first showed a better reproducibility (Supporting Figure S-1). We also determined the optimal incubation time. Different incubation times (5, 10, 15, 20, 30 min at 70 °C) were tested to maximize the intensity of the MS signals and evaluate metabolite degradation (Supporting Figure S-2). The signals were stable for all compounds and extraction times, indicating no significant degradation occurred in our conditions. Based on these results, we selected an extraction time of 10 min.

Finally, we implemented the isotope dilution mass spectrometry (IDMS) approach to ensure accurate absolute quantification of the different metabolites (Wu et al. 2005). The fully ^13^C-labeled cellular extract used as internal standard was produced from a *S. cerevisiae* strain with high pools of isoprenoids precursors (strain S037) grown on U-^13^C-glucose as sole carbon source, and using the optimized sampling and extraction procedures. All intermediates showed a high ^13^C-incorporation (mean molecular ^13^C-enrichment > 98%).

### Method validation in cellular extracts

We evaluated the LC–HRMS method by preparing and analyzing in triplicate a mixture of commercial standards (at concentrations from 0.08 nM to 10 µM) prepared in a biological matrix (a uniformly ^13^C-labeled extract used as internal standard), and following the Metabolomics Standards Initiative guidelines (Sumner et al. 2007). The method showed an excellent stability in retention time (within ± 5%) for all metabolites, and the instrument remained stable without the need for extra cleaning or maintenance. We estimated the limit of detection (LOD), limit of quantification (LOQ) and linearity according to the Eurachem guidelines (Örnemark and Magnussonm 2014), with an acceptable accuracy and precision threshold fixed at ± 20%. Results (Table 1) show a high repeatability and reproducibility for all concentrations, with an excellent overall correlation coefficient (R^2^ > 0.99) for all metabolites. LOD was below 0.1 pmol injected on column for most compounds, and LOQ was about 0.1–1.5 pmol over a range of concentration spanning two to three orders of magnitude. Intra- and inter-day assay reproducibility was below 20% for all compounds. Overall, the method was found to be highly sensitive and reproducible for all compounds tested.

### Determination of concentration and isotopic profiles of isoprenoids precursors

To demonstrate the applicability of the proposed workflow, we carried out functional investigations to guide rational design of phytoene production in *S. cerevisiae*. We first optimized the pool of phytoene precursors in the wild-type (WT) metabolic chassis by constructing the strain S037, which overexpresses HMG-CoA reductase (HMG1t), FPP synthase (ERG20) and GGPP synthase (CrtE). We then constructed the strain S023 by expressing a heterologous phytoene synthase (CrtB from *Pantoea ananatis*) which converts GGPP into phytoene. We performed metabolomics experiments to quantify the concentration of each intermediate in the three strains, and stable isotope labeling experiments which are of greatest interest to measure metabolic fluxes and infer flux control in living cells.

**Fig. 2:**
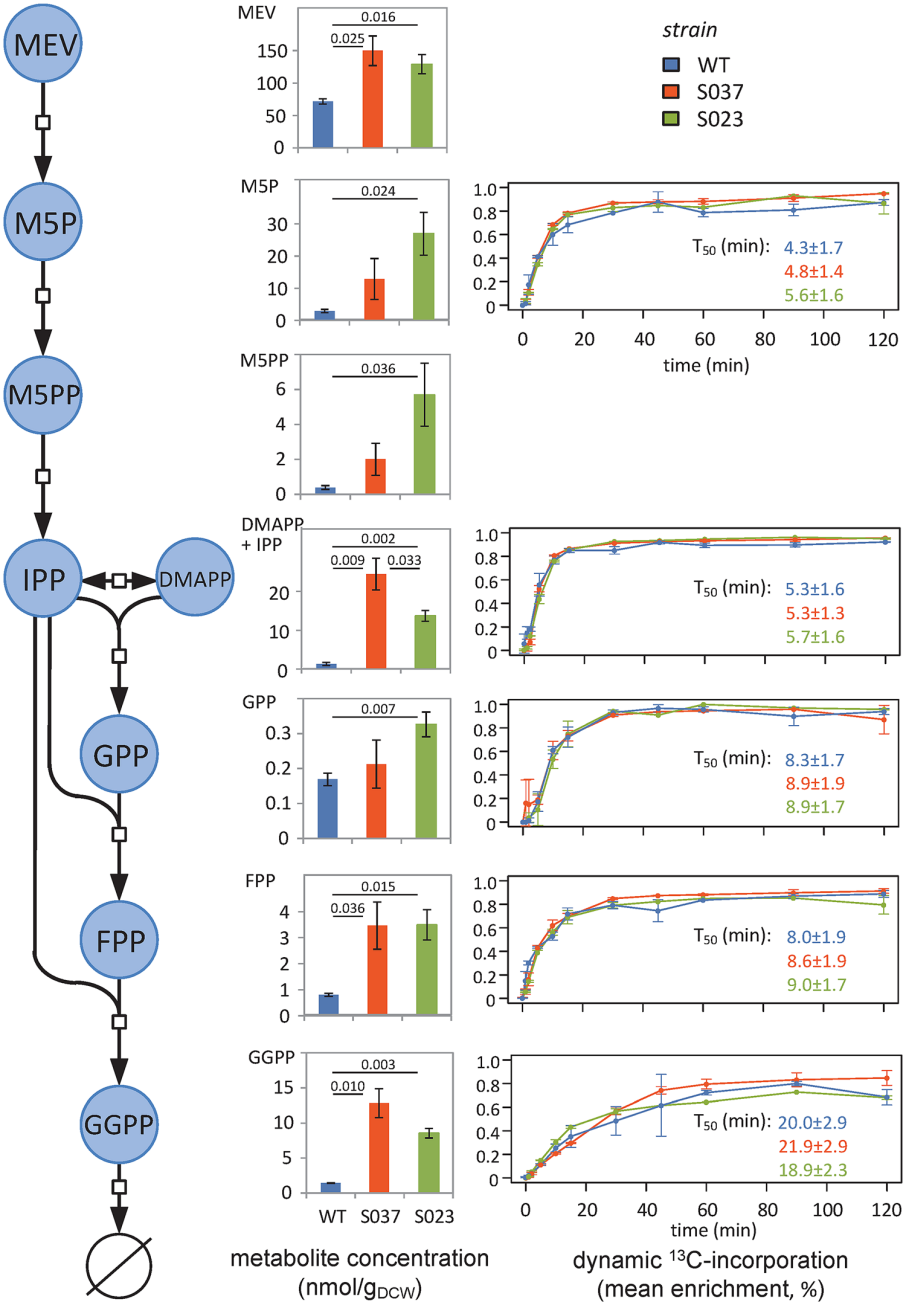
Absolute intracellular concentrations of isoprenoids precursors and dynamic ^13^C-incorporation following a switch from unlabeled to U-^13^C-glucose. A two-sided Welch’s *t*-test was performed to assess the significance of differences observed between strains. For metabolomics experiments, p-values are given when lower than 0.05. For isotope labeling experiments, differences in T_50_ between strains were not significant (p > 0.10)

Metabolomics results showed very different concentration profiles in the three strains, with a good biological reproducibility for all intermediates (average RSD of 20%) (Fig. 2). GGPP concentration was increased tenfold in strain S037 (12.8 ± 2.0 nmol/g DCW) compared to the WT strain (1.4 ± 0.1 nmol/g DCW) (p = 0.010). Other pools of intermediates were also significantly increased in S037 (MEV, p = 0.024; IPP/DMAPP, 0.009; FPP, p = 0.036). This confirms the relevance of overexpressing HMG1t, ERG20 and CrtE to optimize the metabolic chassis. Additional expression of CrtB in strain S023 resulted in a slight decrease of GGPP pool (8.5 ± 0.7 nmol/g DCW, p = 0.056) compared to S037 to support phytoene production (with a total phytoene production of 1.2 ± 0.1 µmol/g DCW).

Fluxes in linear pathways such as the isoprenoids biosynthetic pathway cannot be resolved using well-established stationary ^13^C-isotope labeling approaches. We thus carried out more complex instationary ^13^C-ILEs, where the transient label incorporation—i.e. isotopologue distribution—was measured into the different intermediates during 120 min following a switch of the cells from a cultivation medium with unlabeled glucose to a medium containing uniformly ^13^C-labeled glucose. Compared to metabolomics experiments, the sensitivity and selectivity of MS analyses tend to decrease in ILEs because a single metabolite gives rise to several MS peaks, and their intensity depend on the incorporation of isotopic tracers (e.g. a metabolite with *n* carbon atoms may give rise to *n *+ *1* MS peaks in a ^13^C-ILE) (Heuillet et al. 2018). This was observed for mevalonate and mevalonate-5-pyrophosphate which showed low and variable signals. However, the method was sensitive enough to accurately quantify the isotopologue distributions of all the other intermediates (Figure S-3), including GGPP which contains a high number (20) of carbon atoms and hence gives a very high number of MS peaks. A total of 671 isotopologues were quantified for each strain (61 isotopologues from 6 metabolic pools × 11 time points). The reproducibility of the method was evaluated from the spread of measured isotopologue abundances and was expressed as the mean standard deviation over all the measured isotopologues (Heuillet et al. 2018). The mean precision on isotopologue quantification was 0.027, 0.013, and 0.013 for the wild type, S037 and S023 strains, respectively. Isotopologue distributions were used to calculate the dynamics of ^13^C-molecular enrichments (Fig. 2), which provide a direct readout of the propagation of tracer isotopes through the isoprenoids precursors and facilitate data interpretation. For each metabolite and each strain, the time needed to achieve 50% of final enrichment (T_50_) was estimated by fitting a logistic model (Kiefer et al. 2015). As expected, label incorporation followed the order of intermediates in the pathway, with a T_50_ ranging from 5 min for M5P to 20 min for GGPP (Fig. 2). As also expected, the final ^13^C-enrichments did not significantly differ between intermediates (~ 90%, p > 0.05). More surprisingly, the dynamic ^13^C-enrichments profiles and T_50_ values of all intermediates were also very similar for the three strains (p > 0.05) despite significant changes in pool sizes.

Integration of metabolomics and isotopic datasets may provide quantitative information on the biosynthetic flux of isoprenoid precursors. The labeling dynamics of a given metabolite reflects its turnover rate, which is roughly equivalent to the ratio of the metabolite pool size and the flux through that metabolite pool. The strong increase of pools in S037 and S023 strains compared to the WT strain are expected to slow down the transient ^13^C-incorporation (Buescher et al. 2015). However, the ^13^C-enrichment profiles and turnover rates were very similar in the three strains, which suggest that the flux increases roughly proportionally with the pools. The relative increase in GGPP biosynthesis in strains S037 and S023 compared to the wild-type strain could thus be estimated from the corresponding change in GGPP concentration. Assuming a constant turnover of GGPP, its biosynthetic flux increased by a factor 9.0 ± 2.6 in strain S037 and 7.0 ± 1.6 in S023 compared to the wild-type strain (p < 0.01), which support the relevance of the proposed strategy to enhance phytoene production.

Inferring absolute flux values from these data would require more sophisticated mathematical models of isoprenoids biosynthesis, such as non-stationary ^13^C-flux models. Still, these results already demonstrate the applicability of the proposed workflow to infer flux information on this pathway in yeast. Concentrations and isotopic labeling of the prenyl pyrophosphate intermediates could also complement other biochemical datasets (e.g. proteomics or enzyme activities) to infer quantitative information on the control exerted by each reaction step on the pathway flux, and thereby drive further strain optimization strategies.

## Conclusion

We presented a workflow for functional analysis of isoprenoids biosynthesis in yeast. Procedures to quench metabolism and extract metabolic intermediates were optimized and a novel method was developed to measure concentrations and isotopic profiles of isoprenoids precursors by high pressure liquid chromatography coupled to high-resolution mass spectrometry. This workflow provides accurate quantification (RSD < 20%) over a large linear range (from 1 to 50 pmol) and showed a high sensitivity (LOD < 0.1 pmol) for all compounds tested. Integration of metabolomics data with dynamic ^13^C-incorporation provides quantitative information on the biosynthetic flux of phytoene precursors in *S. cerevisiae*. Overall, these results illustrate the value of the present workflow during iterative construction of a phytoene producing strain in biotechnology.

This methodology closes one of the remaining gaps for comprehensive, quantitative understanding of isoprenoids biosynthesis. Access to absolute intracellular concentrations of all intermediates in combination with the ability to quantify their isotopologue distribution will be a valuable tool for future investigations. It may also support the development of kinetic models of isoprenoids metabolism, thus opening the way to comprehensive, mechanistic understanding of the control and regulation of their biosynthesis. This workflow was designed and validated for yeast but should be applicable to other eukaryotic and prokaryotic organisms, including bacteria, plants, and mammals, after adaptation of organism-dependent steps (in particular quenching and extraction).

## Electronic supplementary material

Below is the link to the electronic supplementary material.

Supplementary material 1 (PDF 775 kb)

## Data Availability

The datasets generated during the current study are available in the MetaboLights repository with identifier MTBLS923 (https://www.ebi.ac.uk/metabolights/MTBLS923).
