# Supplementary material for: Functional analysis of isoprenoid precursors biosynthesis by quantitative metabolomics and isotopologue profiling
Source: Metabolomics. 2019 Aug 21;15(9):115. doi: 10.1007/s11306-019-1580-8 (PMC6704079; doi:10.1007/s11306-019-1580-8)

Supporting information

Functional analysis of isoprenoid precursors biosynthesis in yeast by quantitative metabolomics and isotopologue profiling

Sara Castano-Cerezo[$,1], Hanna Kulyk-Barbier[$,1], Pierre Millard[$,1], Jean-Charles Portais[1,2], Stéphanie Heux[1], Gilles Truan[1], Floriant Bellvert[1,2,*]

[1]LISBP, Université de Toulouse, CNRS, INRA, INSA, Toulouse, France
[2]MetaToul-MetaboHUB, National infrastructure of metabolomics and fluxomics, Toulouse, France

[$]co-first authors

*corresponding author: floriant.bellvert@insa-toulouse.fr

Table of contents

Table S-1. Comparison of available methods for quantification of isoprenoids precursors.

Table S-2. Strains used this study.

Figure S-1. Optimization of the extraction procedure.

Figure S-2. Determination of optimal incubation time for extraction of isoprenoids precursors.

Figure S-3. Dynamic ^{13}C-incorporation through the isoprenoid biosynthetic pathway.

Table S-1. Comparison of available methods for quantification of isoprenoids precursors. Limit of detection for all the isoprenoids precursors are provided in pmol of metabolite injected. *na: not available. *compounds co-elute and cannot be separated.*

DOI	Derivatization	Detection	Sample	Run (min)	Limit of detection (pmol injected)							
					MEV	M5P	M5PP	IPP	DMAPP	GPP	FPP	GGPP
10.1016/j.ab.2008.04.021	Yes	Fluorescence	Mouse tissue	21	-	-	-	-	-	-	0.1	0.1
10.1016/j.ab.2008.08.023	No	MS (MRM mode)	HepG2	12	0.01	0.01	0.01	1*	0.3*	0.3	0.3	0.6
10.1371/journal.pone.0049004	No	MS	*Saccharomyces pombe*	16	-	-	-	-	-	-	0.5	0.5
10.1104/pp.16.01392	No	MS	*Arabidopsis thaliana*	30	-	-	-	-	-	*na*	*na*	*na*
10.1007/s00216-017-0293-y	No	MS (MRM mode)	Human plasma	15	-	-	-	-	-	-	0.5	*na*
10.1016/j.chroma.2017.01.084	No	MS (SIM mode)	Blood serum	11	-	-	-	-	-	-	0.1	3
10.3390/ijms11103965	No	MS (MRM mode)	Mouse brain	16	-	-	-	-	-	-	-	*na*
10.1007/s00216-008-2306-3	Yes	Fluorescence	Human brain tissue	20	-	-	-	-	-	-	*na*	*na*
10.1016/j.jchromb.2009.07.010	No	MS	Human breast cancer cells	13	-	-	-	1.3*	1.3*	-	-	-
10.1093/femsyr/fox032	No	MS	*Saccharomyces cerevisiae*	15	-	-	-	-	*na*	*na*	0.01	-
10.1016/j.ab.2011.12.037	No	MS (MRM mode)	*Arabidopsis thaliana*	22	-	-	-	-	-	0.9	1.1	1.9
10.1016/j.chroma.2018.05.006	No	MS (MRM mode)	Natural rubber latex	12	-	-	-	-	0.1	-	0.04	-
10.1074/jbc.M109.083931	No	MS (MRM mode)	Mammalian cells	10	-	-	-	-	-	*na*	*na*	*na*
10.1007/s11745-009-3355-x	Yes	Fluorescence	Mammalian cells	28	-	-	-	-	-	0.02	-	-
10.1006/abio.1997.2314	Yes	Fluorescence	Dog and human plasma	20	-	-	-	-	-	-	0.01	-

Table S-2. Strains used this study.

Strain	*Genotype*	*Plasmid*
Wild type	CEN.PK2-1 MATa; his3D1; leu2-3_112; ura3-52; trp1-289; MAL2-8c; SUC2	pFL36 (LEU2)
S023	CEN.PK2-1 MATa; his3D1; leu2-3_112; ura3-52; trp1-289; MAL2-8c; SUC2<br>bts1::ADH1t-HMG1t-TDH3-PGKp-ERG20-ADH2t-URA3,<br>X-2::*PDC1p- CrtB* $^{P.\ ananatis}$*-CYC1t-TRP1,*<br>X-4:: *HIS3*	pENZ017<br>TEF1p-CrtE$^{X.dendrorus}$ -ADH1t (LEU2)
S037	CEN.PK2-1 MATa; his3D1; leu2-3_112; ura3-52; trp1-289; MAL2-8c; SUC2<br>bts1Δ::ADH1t-HMG1t-TDH3-PGKp-ERG20-ADH2t-URA3,<br>x-2:: TRP1,<br>x-4:: HIS3	pENZ017<br>TEF1p-CrtE$^{X.dendrorus}$ -ADH1t (LEU2)

Figure S-1. Optimization of the extraction procedure. Samples (10 mL) were collected from a unique cultivation by fast filtration, extracted using different procedures, and analyzed by LC-HRMS. The extraction solution providing the most intense and reproducible signals was isopropanol/H_2O + 100mM NH_4CO_3 (1:1).

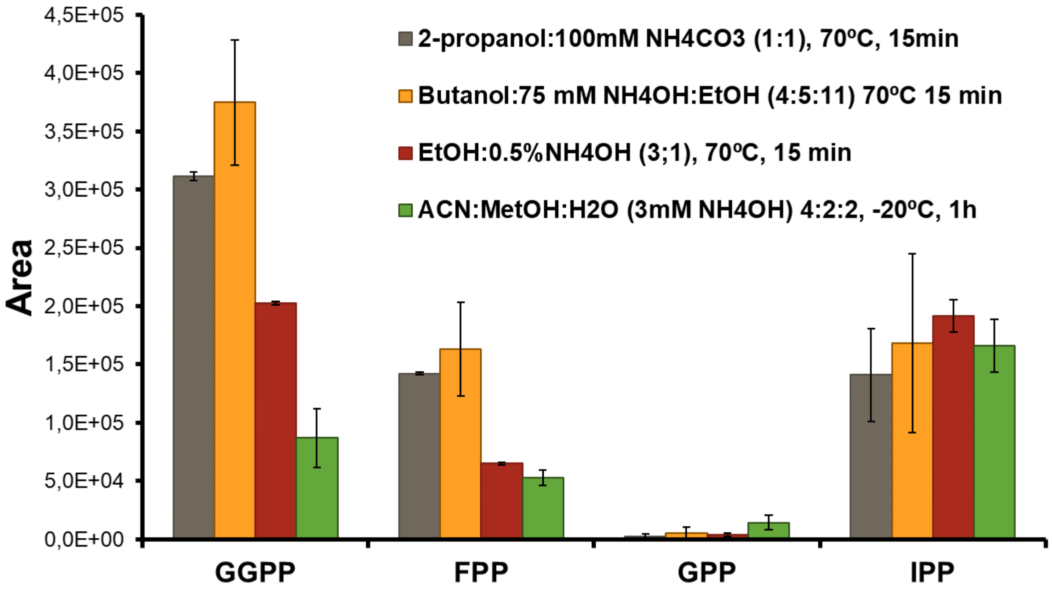

Figure S-2. Determination of optimal incubation time for extraction of isoprenoids precursors. Samples (10 mL) were collected from a unique cultivation by fast filtration, incubated in the extraction solution (isopropanol/H_2O + 100mM NH_4HCO_3, 1:1) at 70°C for 2, 5, 10, 15, 20 or 30 minutes, and cooled on ice before adding the U-^{13}C-internal standard. The concentration of all compounds were stable for all extraction times, indicating no significant degradation occurred in our conditions.

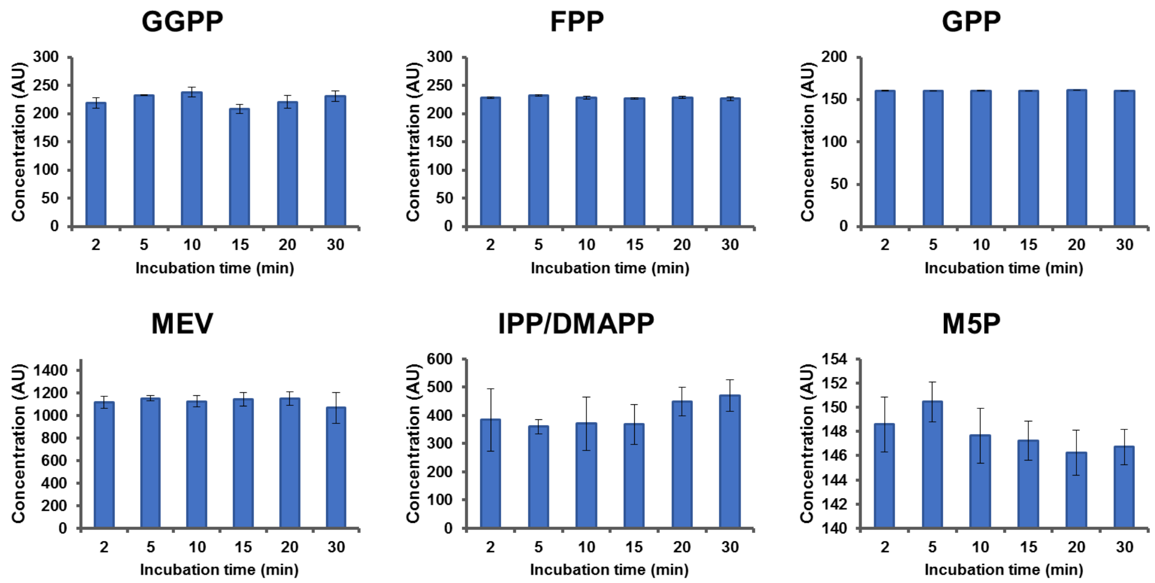

Figure S-3. Dynamic ^{13}C-incorporation through the isoprenoid precursors pathway. Isotopologue distributions of the mevalonates and prenylpyrophosphates intermediates were measured during 120 min following a switch from unlabeled to U-^{13}C-glucose in strains WT, S037 and S023.

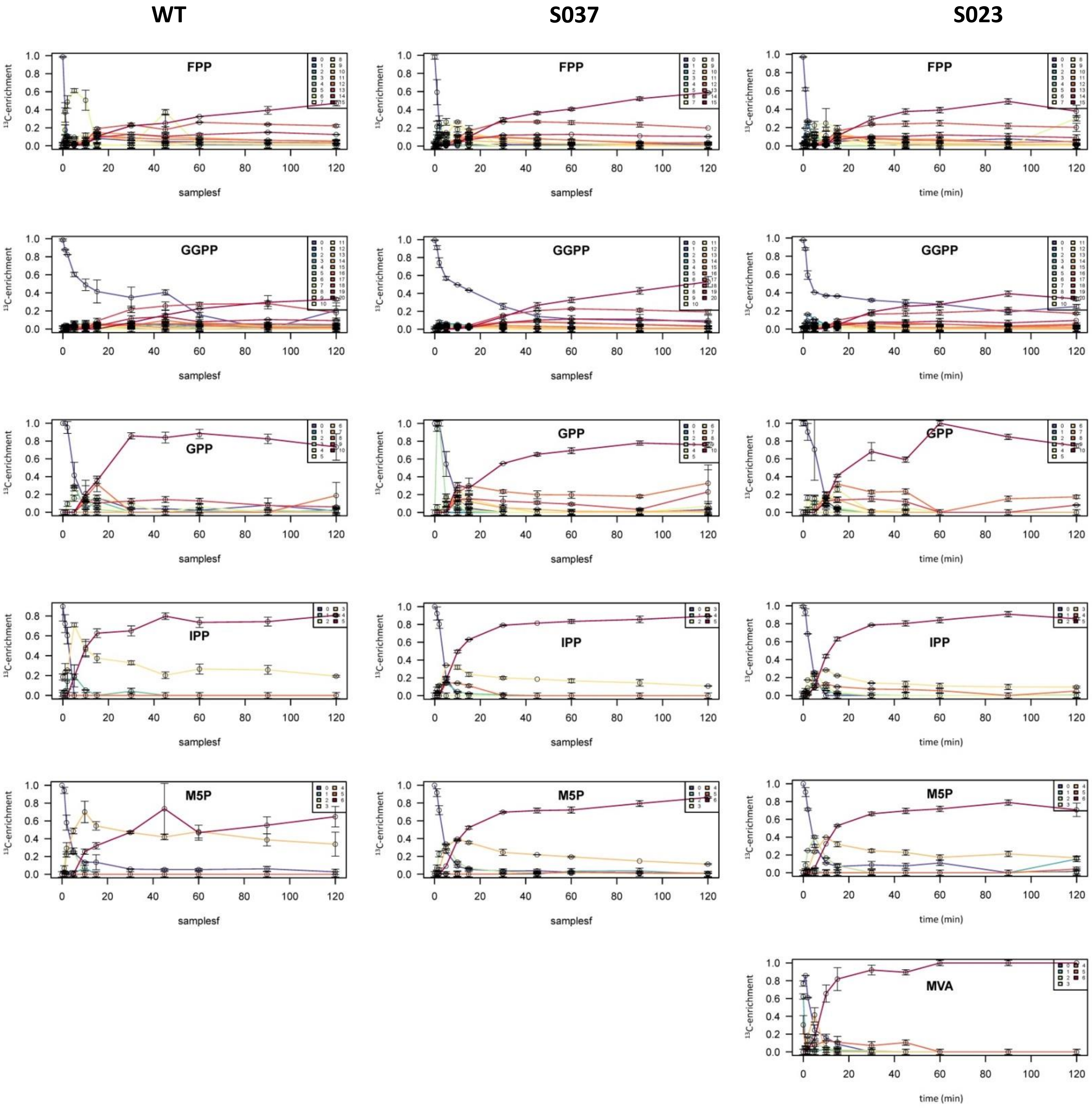

Supplement: Supplementary file 1 — Supplementary material 1 (PDF 775 kb) [file 11306_2019_1580_MOESM1_ESM.pdf]
